# Mechanism and role of nuclear laminin B1 in cell senescence and malignant tumors

**DOI:** 10.1038/s41420-024-02045-9

**Published:** 2024-06-01

**Authors:** Tingcong Lv, Cong Wang, Jialin Zhou, Xiao Feng, Lijun Zhang, Zhe Fan

**Affiliations:** 1 Department of General Surgery, the Third People’s Hospital of Dalian, Dalian Medical University, Dalian, China; 2 Department of General Surgery, The Second Affiliated Hospital of Dalian Medical University, Dalian, China; 3 School of Chemistry, Dalian University of Technology, Dalian, China; 4 Liaoning Province Key Laboratory of Corneal and Ocular Surface Diseases Research, the Third People’s Hospital of Dalian, Faculty of Medicine, Dalian University of Technology, Dalian, China

**Keywords:** Oncogenes, Oncogenes

## Abstract

Nuclear lamin B1 (LMNB1) is a member of the nuclear lamin protein family. LMNB1 can maintain and ensure the stability of nuclear structure and influence the process of cell senescence by regulating chromatin distribution, DNA replication and transcription, gene expression, cell cycle, etc. In recent years, several studies have shown that the abnormal expression of LMNB1, a classical biomarker of cell senescence, is highly correlated with the progression of various malignant tumors; LMNB1 is therefore considered a new potential tumor marker and therapeutic target. However, the mechanism of action of LMNB1 is influenced by many factors, which are difficult to clarify at present. This article focuses on the recent progress in understanding the role of LMNB1 in cell senescence and malignant tumors and offers insights that could contribute to elucidating the mechanism of action of LMNB1 to provide a new direction for further research.

## Facts

- LMNB1 is a protein with functional activity that affects changes in the chromatin state. It is involved in maintaining the structural stability of the nucleus and undergoes dynamic changes during the cell senescence program.
- Recent findings show that LMNB1 protein dysfunction interferes with the cellular senescence process, which is highly associated with tumor progression.
- LMNB1 expression levels vary in different malignant tumors, and the mechanism of action involved in cellular senescence for promoting malignancy has not yet been fully clarified.

## Open Questions

- Can LMNB1 be used as a stable specific marker in malignancies promoted by age-related factors?
- Is the mechanism of action of targeting LMNB1 more conducive to malignancy prevention or treatment?
- Whether the expression level of LMNB1 can serve as a prognostic indicator of related malignant tumors and provide reference for the survival assessment of patients?

## Introduction

Cellular senescence is a multifactorial process that occurs throughout an organism’s lifetime, and it is characterized by the loss of cellular and tissue integrity, resulting in biological dysfunction and an increased risk of age-related diseases, particularly malignancies [1]. Following the increase in human life expectancy and adoption of sedentary lifestyle and unhealthy eating habits, the incidence of malignant tumors is increasing each year, with a trend of gradual decrease in the age of onset. A 2022 analysis of the global cancer data reported that cancer was a major public health issue worldwide and the second leading cause of death in the United States, wherein nearly 1,918,030 new cases of cancer and 609,360 deaths associated with cancer were predicted by The American Cancer Society in 2022 [2]. Targeted prevention before cancer occurrence, accurate diagnosis, effective treatment at the early stage of the disease, and accurate evaluation after treatment can significantly reduce the incidence and recurrence rate and improve the survival time of cancer patients.

**Fig. 1:**
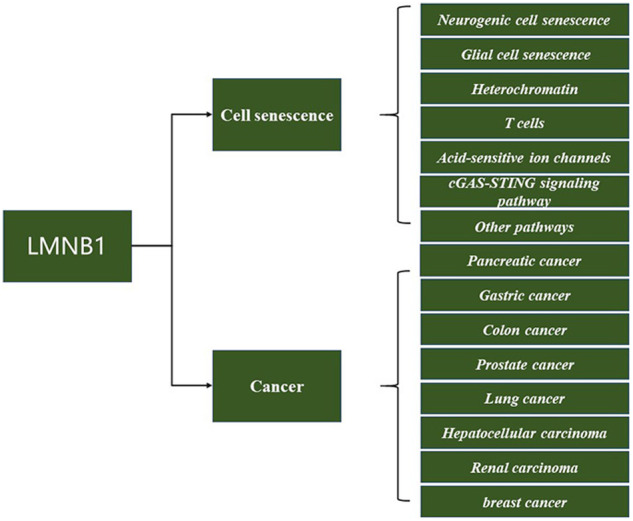
Summary diagram. Involvement of LMNB1 in cell senescence and cancer.

Lamin, a type of fibrin, is a core regulator role in mediating the structure and function of the nucleus [3]. Two types of lamins have been identified: type A and type B. In recent years, in-depth studies have shown that the protein nuclear lamin B1 (LMNB1) was closely associated with human diseases such as cell aging and malignant tumors, and traces of LMNB1 have been detected in multiple tumor sites in the human body (Fig. 1). Some progress has also been made in the clinical application of LMNB1, and the findings indicate that LMNB1 could become a new tumor marker and therapeutic target [4]. The present article reviews the current progress in the research on the relationship between LMNB1 and cell senescence and malignant tumors, with a hope to provide a reference for the diagnosis and treatment of diseases and to generate new ideas for further research.

## Overview of lamins

**Fig. 2:**
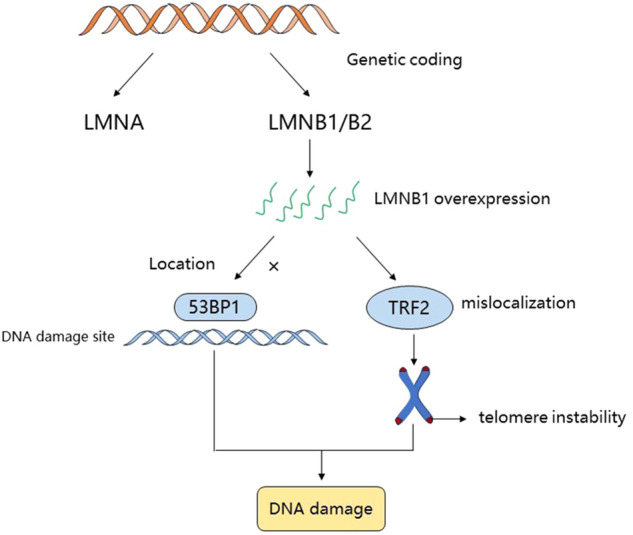
Mechanism of action of LMNB1 leading to DNA damage. LMNB1 can induce telomere instability through mislocalization of TRF2, prevent 53BP1 from recruiting to the corresponding DNA damage site, resulting in persistent DNA damage, defective non-homologous end joining, and increased sensitivity to double-strand breaks.

The nuclear lamina is a protein network composed of nuclear lamins. According to previous literature, mammalian laminin is encoded by three lamin genes: lamin-A (*LMNA* encoding lamin A and lamin C) and lamin-B (*LMNB1* encoding lamin B1 and *LMNB2* encoding lamin B2 and its splicing variant lamin B3) [5, 6]. Laminin plays an important role in composing nuclear structure, regulating chromatin distribution, and ensuring gene expression and DNA replication and repair; thus, it responds to cell cycle progression, stress, and cell proliferation and differentiation [7]. Recent studies have reported that LMNB1 was associated with cell senescence and multiple malignancies and may predict poor survival outcome of cancer patients [8]. Pennarun et al. [9] found that LMNB1 was a new interacting partner of TRF2, and LMNB1 overexpression induced telomere instability through mislocalization of TRF2. LMNB1 overexpression also prevents 53BP1 from properly recruiting to the corresponding DNA damage site, resulting in persistent DNA damage, nonhomologous end junction defects, and increased sensitivity to double-strand breaks [10]. The mechanism of action of LMNB1 is shown in Fig. 2.

## LMNB1 and cancer senescence

Cell senescence is a fundamental aging process that plays a critical role in driving these changes [11]. Cell senescence is also a permanent nondividing cell state triggered by several stressors, which activates the tumor suppressor pathways p53/p21WAF1 and p16INK4a/pRB and induces significant changes in the cell. Changes in senescent cells can be specific to chromatin remodeling, metabolic switching, and altered gene expression and regulate the secretion of inflammatory cytokines and growth factors and production of proteases and other senescence-related secretory phenotype (SASP) molecules [12]. In general, senescent cells exert a potential profibrotic effect, resulting in organ fibrosis and eventually leading to disease development [13]. Therefore, it is crucial to assess the factors related to LMNB1-associated cell aging (Table 1).

**Table 1:** Relationship between LMNB1 and cellular senescence.

Associated cells and influencing factors	Mechanism	Effects on cellular senescence	References
NSCs	Deletion of the LMNB1 gene leads to depletion of NSCs	facilitate	[18]
Glial cells	LMNB1 level is normal or decreased in glial cells	facilitate	[22–24]
Heterochromatin	Heterochromatin loss leads to telomere shortening and induces DNA damage, which in turn leads to the release of KAP1 and LMNB1	facilitate	[12, 25]
T cells	DNA hypomethylation and LMNB1 defects may lead to T cell-related sclerosis	facilitate	[26]
Acid-sensitive ion channels	ASIC1a induces a decrease in LMNB1	facilitate	[27]
CCF-cGAS-STING signaling pathway	Curcumin inactivates the CCF-cGAS-STING pathway by blocking the interaction between light chain β and LMNB1	restrain	[31]
Doxycycline	Doxycycline restores the levels of LMNB1 by decreasing the degree of mTOR and NF-κB activation	restrain	[33]

### Relationship between LMNB1 and neurogenic cell senescence

Lamin is an important structural component of the nuclear membrane. It binds to heterochromatin for promoting its distribution within the nuclear membrane and interacts with epigenetic modifiers to control gene transcription [14]. Most studies have shown that LMNB1 was a key nuclear envelope protein [7] that was downregulated in senescent cells. LMNB1 downregulation can be detected at both in vivo and in vitro mRNA levels [3] and leads to decreased nuclear membrane integrity [3]; this phenomenon is used as a marker of cell senescence [15–17]. Two recent reports have found that LMNB1, as one of the epigenetic factors of neural stem cells (NSCs), is involved in the regulation of their cell senescence process [18]; the studies further showed that the deletion of the *LMNB1* gene can result in the final outcome of differentiation and long-term depletion of the NSC pool [18]. An in vitro study also supported the finding of age-related decreased expression of LMNB [19]; the approach used for reversing the age-related decreased expression of LMNB1 in NSCs induces cell proliferation, thereby increasing the level of adult neurogenesis in the aging hippocampus [19]. The decrease in hippocampal LMNB1 expression during aging is limited to neurogenic lineages and does not occur in astrocytes, microglia, and interneurons [20].

### Relationship between LMNB1 and glial cell senescence

Aging causes morphological transformation of human microglia; this condition is termed dystrophic microglia. Neumann et al. [21] tested whether malnourished microglia displayed characteristic markers of cellular aging by double and triple staining of temporal lobe and brain stem slices from 14 individuals. They found no reduction in LMNB1 expression in malnourished microglia or around the laminae of branched microglia nuclei in the same microscopic field. This result was inconsistent with the deletion or morphological change in LMNB1 observed in previous rodent aging models [22, 23]; this might be because, in addition to the mechanism regulating cell aging, various pathogenic mechanisms induce the transformation of microglia in malnutrition. Matias et al. [24] examined astrocytes under in vitro conditions, in the brains of elderly mice, and in the brain tissues of deceased elderly people; they found that a significant loss of LMNB1 expression was a sign of aging astrocytes. The phenomenon of severely reduced LMNB1 expression was observed in the hippocampal granulosa layer of the brain in older departed humans without dementia as well as in the dentate gyrus (including hippocampal astrocytes) in aging mice [24]. However, the mechanism of aging remains unclear and requires further investigations.

### LMNB1 affects cell senescence through heterochromatin

Among the several changes that occur in chromatin structure during cell senescence, the dynamics of heterochromatin are complex, and the constitutive heterochromatin is lost in most cells, resulting in its transcriptional repression and leading to aging [25]. Mendeous-Bermudez et al. [12] studied the relationship between telomere shortening and constitutive heterochromatin loss, two key events in aging; they observed that the programmed mechanism of TP53 gene activation was charged to the seemingly chaotic event of constitutive heterochromatin loss during the cell senescence process [12]. TRF2 downregulation in senescent cells triggers the breakdown of pericentromeric heterochromatin (PCH) in an ATM-dependent manner [12]. This observation, combined with the fact that TRF2- and ATM-dependent KAP1 and LMNB1 association mechanisms regulate PCH, suggests that aging-induced dispersion of PCH concentration involves KAP1 and LMNB1 release activity in the corresponding region [12]. KAP1 and LMNB1 are released from PCH following a DNA damage response induced by telomere shortening in replicative senescent cells, while the expression of the TRF2 allele lacking the N-terminal domain leads to the loss of PCH protection but maintains telomere stability. Interventions involving TRF2 will prevent heterochromatin breakdown during aging, thus providing a valuable strategy to prevent age-related diseases, including cancer.

### LMNB1 affects cell senescence through T cells

Age-related changes in T cell function play a central role in immune aging. The role of aging in the reduction of the T cell pool (mainly due to thymus degeneration) has been extensively studied. There is, however, a growing body of evidence that the relative nuclear size influences changes in the hardness of T cells [26]. Gonzalez-Bermudez et al. [26] studied age-related changes in T cell migration and indicated that intercellular variation directly occurs due to the aging process, and aging is related to the evolution of the relative size of the nucleus. Changes in the relative size of the nucleus due to DNA hypomethylation and LMNB1 defects may lead to the loss of migration potential at a higher age, eventually leading to T cell-related sclerosis.

### LMNB1 affects cell senescence through acid-sensitive ion channels

Ding et al. [27] studied the mechanism of acid-sensitive ion channel 1a (ASIC1a) in chondrocyte senescence and osteoarthritis (OA). The results show that ASIC1a, as a rich proton-activated cation channel in chondrocytes, can not only act as a perceptron to accept pH changes in the joint cavity but also regulate its changes simultaneously [27]. In addition, LMNB1 can be used as a substrate for autophagy, which is induced by ASIC1A-mediated autophagy pathway-dependent protein degradation; in the OA rat model, blocking ASIC1a can protect cartilage tissue, restore LMNB1 expression level, and inhibit chondrocyte senescence [27].

### LMNB1 affects cell senescence through the cGAS-STING signaling pathway

**Fig. 3:**
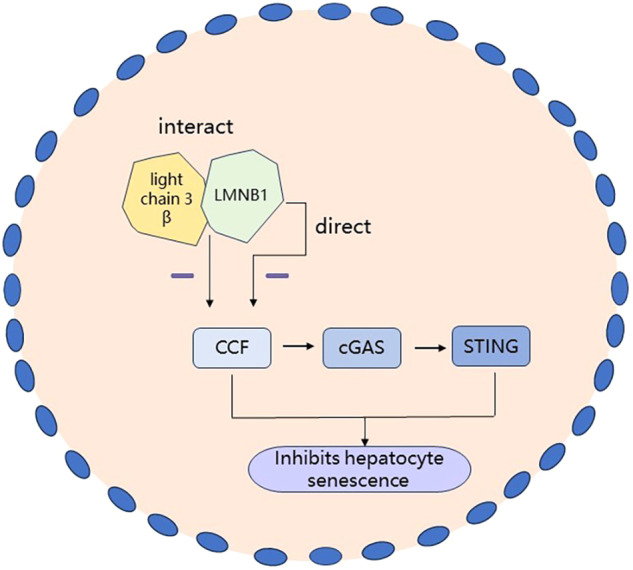
Mechanism of attenuation of hepatocyte senescence by the CCF-cGAS-STING signaling pathway. Interaction of light chain 3 β with LMNB1 or direct transcriptional regulation of LMNB1 inhibits the CCF-cGASSTING pathway to suppress hepatocyte senescence.

Recent studies have tended to focus on the important role of hepatocyte senescence in the alcoholic fatty liver disease (AFLD) progression, and the results suggested that inhibiting hepatocyte senescence may be an effective intervention and treatment strategy for AFLD [28, 29]. Qi et al. [30] analyzed the effect of curcumin on alleviating AFLD and the associated mechanism by regulating the cytoplasmic chromatin fragment-cyclic guanosine phosphate-adenylate synthase-membrane protein interferon stimulating factor (CCF-cGAS-STING) signaling pathway related to hepatic cell aging (Fig. 3). The results showed that curcumin inhibited hepatocyte senescence and alleviated AFLD by blocking the interaction between light chain 3 beta and LMNB1 and inactivating the CCF-cGAS-STING pathway. However, this study did not provide direct evidence of a causal relationship between hepatocyte senescence and hepatic steatosis; hence, further studies are required to validate this relationship. Dou et al. [31] suggested that the silencing of the cGAS-STING pathway effectively reduced the NF-κB-mediated SASP program. However, the effect of silencing this pathway on γ-H2AX, aging-related heterochromatin, or CCF was low in senescent cells. Sladitschek-Martens et al. [32] noted that the regulatory effect of the cGAS-STING signaling pathway partly depended on the direct transcriptional regulation of LMNB1, and the inhibition of the STING signal could be effective in limiting cell senescence-related inflammation and suppressing the aging process.

### LMNB1 affects cell senescence through other pathways

The increased risk of cardiovascular diseases caused by excessive alcohol consumption might be associated with accelerated cellular aging. Li et al. [33] studied the damages caused by ethanol in human vascular diseases; the results showed that doxycycline restored the level of aging-related proteins such as LMNB1 by reducing the extent of mTOR and NF-κB activation, thus alleviating ethanol-induced inflammation and aging. By observing the changes of mouse lung epithelial cells and primary human small airway epithelial cells after treatment with a high concentration of oxygen (95% O_2_/5% CO_2_) or air (21% O_2_/5% CO_2_) for 24 h. Maeda et al. [34] found that high oxygen concentration induced senescence by increasing the level of miR-34a-5p. Moreover, *LMNB1* gene deletion increased *p21* gene expression. Recent studies have shown that oleuropein aglycone and hydroxytyrosol played a protective role in 8-Gy radiation-induced aging by maintaining LMNB1 expression and reducing the age-related SASP, which improved the efficacy and safety of cancer radiotherapy [35].

## LMNB1 and malignant tumors

In the cell nucleus of higher animals, the nuclear fiber layer is scattered throughout the inner nuclear membrane and can interact with chromatin. It can also bind to transcription factors, which in turn regulate gene transcription and DNA replication, and help maintain nuclear integrity [36]. In living organisms, micronucleus forms around chromosomes or fragments of chromosomes that inadvertently separate and recruit their own nuclear membranes during mitosis [36]. Micronucleus is a biomarker of chromosomal instability in cancer; it frequently appears during early human embryogenesis and occurs less frequently in healthy tissues [37]. Micronucleus size is closely associated with the LMNB1 level and pore density in intact micronucleus. Interestingly, Mammel et al. [38] indicated that the LMNB1 level could not fully predict laminar tissue or membrane stability. The unknown factors associated with gene density are thought to have a different function, wherein they inhibit the emergence of the nuclear laminar space and delay membrane rupture until the late phase in the cell cycle [38]. Although micronucleus is a well-known biomarker of genotoxic damage, little is known regarding the biological consequences of micronucleus induction. Reimann et al. [39] found that the micronuclei did not share a common fate after treating HeLa cells with different genetic agents; however, DNA stability and micronucleus envelope were impaired, and LMNB1 was expressed in approximately 50% of the micronuclei. This finding indicated that micronucleus breakdown is required for chromosome fragmentation, while the damaged DNA is retained in the cell, which may promote tumor development [39]. Nuclear membrane rupture is known to be associated with the loss or defect of lamin; however, the key physical determinants of nuclear membrane rupture remain unknown. Some researchers believe that high nuclear membrane curvature is related to its rupture [40]. A previous study showed that isolated micronuclei lacked functional LMNB1 and were more prone to envelope rupture, resulting in DNA damage, abnormal replication, and even cancer development [41]. The increased expression levels of LMNB1 can enhance cell migration potential, which may contribute to the progression and metastasis of some cancers [42, 43], including pancreatic cancer (PC), prostate cancer, lung cancer, hepatocellular carcinoma (HCC), and kidney cancer. Hua et al. [44] verified that the high LMNB1 expression is necessary in most cancers by performing weighted gene co-expression network analysis and disease-free survival analysis. Moreover, the LMNB1 expression level was significantly correlated with prognosis in nearly 50% of the cancer cases studied [44]. The authors also concluded that the function of the LMNB1 protein affects mRNA binding, olfactory transduction, gene silencing and other functional mechanisms in some cancers, and immune cell activities, such as the infiltration level of macrophage M1 and the activation of CD4+ memory T cells, are also involved in the expression of LMNB1 [44]. Another study reported that the inhibition of LMNB1 expression induced apoptosis by regulating cell cycle, cell migration, and cell proliferation in in vitro experiments and in mouse cancer models and weakened its invasive ability [45]. Interestingly, in breast cancer, a low LMNB1 expression level was associated with a poor clinical outcome [46]; this result was in contrast to that observed in other tumors with low LMNB1 expression level. Mixed results were also observed for stomach cancer and colorectal cancer. Thus, to a certain extent, LMNB1 is specifically expressed among various cancer types [47]. Hence, it is necessary to evaluate the expression level of LMNB1 in different malignant tumors.

### LMNB1 and pancreatic cancer

Pancreatic cancer (PC) is also known to be a fatal malignant disease [48], and patients who are not effectively predicted by current biological markers and are clinically diagnosed are often in advanced stages and have the highest proportion of cancer-related deaths and cancer aggressiveness [49]. Therefore, the search for effective biomarkers is crucial for the early detection of PC [49]. Nuclear organization and structure are critical for maintaining genome integrity and for epigenetic regulation and gene expression [50]. Following chromosomal rearrangement and altered gene expression, the loss of LMNB1 is observed in human laminosis and sporadic cancer. Earlier studies have shown that the targeted transcription factor Sp1 was critical in regulating LMNB1 expression in PC cells [51]. Through Sp1, LMNB1 overexpression in PC cells not only greatly enhances cell adhesion but also promotes independent growth, and cell migration [52]. Interestingly, Li et al. [53] conducted RT-PCR and western blotting analysis to measure the extent of LMNB1 expression in PC cells and reported that the expression of LMNB1 in PC cells was abnormally upregulated; the LMNB1 expression level was higher in poorly differentiated or metastatic PC cells. Following the knockout of the *LMNB1* gene in AsPC-1 and PANC-1 cells, the results showed that mitosis of PC cells was blocked in the G1 phase, with lower metastasis and invasion [53]. Betulinic acid (BA) showed an antitumor effect by downregulating LMNB1 expression; however, no correlation was observed between LMNB1 expression and Sp1 signaling [53]. Tumor suppressor p53 has the ability to regulate specific transcriptional programs through lamin A/C, but its relationship with p53 is unclear [53]. Recently, Panatta et al. [54] reported that the loss of p53 was related to the upregulation of members belonging to the nuclear pore complex and directly altered the transcription of LMNB1; this indicates that p53 directly regulates the transcription of LMNB1. The interaction of LMNB with Nup210 shows that some genomic loci influence gene expression and rearrangement of genes in PC cells. LMNB1 has an important effect on the diagnosis and prognosis of PC, and more studies are needed to explore it [53, 54].

### LMNB1 and gastric cancer

As a common malignant tumor in humans, GC is also one of the major causes of cancer-related deaths worldwide [55]. The pathogenetic mechanisms of GC remain unclear. Currently, GC is diagnosed based on alpha-fetoprotein (AFP), cancer antigen 19-9 (CA19-9), cancer antigen 125 (CA125), carcinoembryonic antigen (CEA), and other tumor markers; however, the specificity of these markers is low, and there is a lack of effective targeted therapies [56]. The current chemotherapeutic agents mainly show nonspecific antitumor effects; moreover, their therapeutic effects are very limited and often accompanied by severe side effects. The total effective rate of radical gastrectomy combined with first-line chemotherapy remains less than 50% [55]. Hence, new therapeutic targets are required to be discovered to improve the clinical outcomes of GC patients. Yu et al. [57] evaluated LMNB1 expression in GC tissues of 71 patients by immunohistochemical assay. It was observed that the expression of LMNB1 was impaired in gastric cancer tissues, and LMNB1 downregulation was accompanied with higher clinical stage, invasion depth, lymph node stage, and poor prognosis [57]. Additionally, LMNB1 knockdown promotes the activity of the phosphatidylinositol 3-kinase/phosphatase and tensin congener/protein kinase B (PI3K/PTEN/Akt) and mitogen-activated protein kinase/extracellular signal-regulated kinase (MAPK/ERK) pathways and decreases the expression of p53/p21WAF1/CIP1 [57]. It also showed that LMNB1 can promote GC cell proliferation and migration [57]. However, the effect of LMNB1 overexpression conversely inhibits the proliferation and migration of GC cells through this mechanism.

### LMNB1 and colon cancer

The most common pathological subtype of colon cancer is colorectal adenocarcinoma, which is one of the leading causes of cancer-related mortality in the digestive system [58]. The incidence of colorectal adenocarcinoma has been steadily decreasing following the improvement of diagnostic and therapeutic strategies; reduction of exposure to social risk factors; and monitoring of CEA, carbohydrate antigen 72-4, CA 19-9, and other tumor markers [59]. However, there are currently no effective treatment options for advanced metastatic colorectal adenocarcinoma, and the survival rate remains unsatisfactory. The results of early studies showed that LMNB1 expression was downregulated in colorectal adenocarcinoma cells [60]. Liu et al. [61] studied the effect of β-asaryl ether (1-allyl-2, 4, 5-methoxybenzyl alcohol), a compound of the traditional herb *Acorus*, on tumors by the MTT method; the results showed that β-asaryl ether may induce cell senescence by upregulating LMNB1 expression, thus inhibiting the proliferation of colorectal cancer cells. Izdebska et al. [62] treated the colorectal cancer cell line LoVo with fluorouracil and found that LMNB1 overexpression inhibited the invasion and migration of colon cancer cells by inducing mitosis inhibition, enhancing the adhesion between cells, and limiting cell migration. This result was consistent with the findings of previous studies [62]. LEF1 is overexpressed in colorectal adenocarcinoma and is associated with the occurrence and progression of multiple tumors [63]. Xiao et al. [64] inhibited LEF1 expression in Caco-2 cells, a human cloned colorectal adenocarcinoma cell line, by the shRNA method, and the results of this study supported that LEF1 downregulation suppressed the expression of LMNB1, thus inhibiting the viability and proliferation of colorectal adenocarcinoma cells. The deficiency of LMNB1 also alters chromatin distribution and chromatid position in the nucleus during tumorigenesis [65]. In-depth research on colon cancer revealed that the occurrence and development of colon cancer involves multiple gene mutations [65]. The data obtained through the cBioPortal database showed no significant difference in long-term follow-up results between patients exhibiting overexpression and underexpression of LMNB1 [65]; however, the survival rate of colon cancer patients with LMNB1 overexpression was lower in the first 30 months after diagnosis [62]. Presently, the molecular mechanisms and the key regulatory factors of progression and metastasis of colorectal adenocarcinoma are difficult to elucidate, and more studies are required on these topics.

### LMNB1 and prostate cancer

With an increasing incidence year by year, prostate cancer (PCa) is the leading common cancer in men in the Western world, particularly in economically developed countries [66]. It is estimated that there will be over 1.4 million clinically diagnosed cases of PCa worldwide, which might lead to the death of nearly 370,000 patients [67]. Although Gleason score grading and serum prostate-specific antigen (PSA) are helpful to grade the risk of PC, the prediction of disease progression is not accurate enough, and misdiagnosis frequently occurs. Presently, there are no good biomarkers that can effectively differentiate between potentially aggressive and inert PCa. This result in overtreatment, particularly in the presence of a certain number of patients who can be treated conservatively [68]. Previous studies have shown that lamins, particularly LMNB1, was critical in PCa aggravation, and the upregulation of LMNB1 was associated with PCa metastasis and postoperative recurrence [69]. Yang et al. [70] conducted in vitro cell function tests and found that the Chinese herb *Sophora alopecuroides* L. suppressed LMNB1 expression, thus inhibiting the migration and proliferation of PC-3 cells, a PCa cell line; this provided a new direction for treating PCa. Hong et al. [71] performed immunohistochemical staining of PCa specimens from 143 patients and found that specimens with LMNB1 expression showed stronger staining results and a higher Gleason score, with significant differences compared to other specimens. The authors suggested that there is a potential link between LMNB1 with the early stages of PCa progression, but the malignant potential of tumor-initiating cells may require additional molecular changes to be explained [71]. However, this study did not exclude the influence of clinicopathological parameters, and the findings need to be validated by further investigations using control variables. Luo et al. [72] conducted a cohort study on the relationship between PCa and LMNB1; the results indicated that LMNB1 upregulation promotes cancer metastasis and adverse survival outcomes in patients with primary PCa. Song et al. [73] also pointed out that LMNB1 expression was upregulated in patients with PCa; the authors believed that this upregulation might be due to hypomethylation.

### LMNB1 and lung cancer

Lung cancer is a major cancer in humans, with the highest mortality and morbidity rates globally [74]. Small cell lung cancer and non-small cell lung cancer are two types of lung cancer; the former group accounts for 85–90% of lung cancer cases, while in the latter group, lung adenocarcinoma is the major common type that accounts for almost 50% of lung cancer cases [75]. Although lung adenocarcinomas typically grow more slowly and have a smaller mass than squamous lung cancer with the same period of growth, lung adenocarcinomas tend to metastasize earlier [75]. Presently, CEA and secretory protein can be used to monitor lung cancer. However, these screening biomarkers lack clinical sensitivity and specificity; moreover, the clinical symptoms at the beginning of the disease are not typical [75]. Most patients diagnosed to have lung cancer show advanced or metastatic lung adenocarcinoma [76]. Recent studies have highlighted the close correlation between lung adenocarcinoma tissue and LMNB1 expression [77]. Garvalov et al. and Jia et al. [4, 7] found that LMNB1 expression levels were reduced in lung cancer patients; additionally, the loss of a single LMNB1 allele can activate the RET/p38 signaling pathway through the recruitment of multi-combing inhibitory complex 2 (PRC2), thus promoting the development and metastasis of lung cancer. The researchers suggested that LMNB1 acted as a tumor suppressor in lung cancer. Interestingly, the overexpression of LMNB1 in lung adenocarcinoma was identified to stimulate the proliferation of lung tumor cells through the protein kinase B (AKT) pathway [78]. Previous studies revealed that lung adenocarcinoma cells showed LMNB1 overexpression, and the knockout of LMNB1 in these cells reduced their growth rate and colony formation ability [79]. Li et al. [80] estimated LMNB1 knockdown in A549 and NCI-1299 lung adenocarcinoma cell lines to investigate the altered cell proliferation after LMNB1 inhibition and to determine the potential underlying mechanism. The results showed for the first time that persistent loss of LMNB1 in lung adenocarcinoma cells induced telomere shortening, DNA damage, cell cycle arrest in the G2/M phase, apoptosis, thus worsening senescence and inhibiting the proliferation of lung adenocarcinoma cells [80]. Tang et al. [45] reported that the knockdown of LMNB1 downregulated the cancer-related proteins CCND1, CDK6, and PIK3CA. Therefore, LMNB1 has excellent potential to function as an indicator for evaluating the clinical prognosis of patients with lung adenocarcinoma and as a target for precise treatment. This result is different from those of previous studies, and further research is required to confirm the study findings.

### LMNB1 and hepatocellular carcinoma

HCC is the sixth most common malignancy worldwide and the third leading cause of cancer-related death [67]. Currently, AFP shows high sensitivity and specificity to detect HCC patients. But the actual clinical results showed that approximately one-fifth of patients with advanced HCC did not exhibit abnormal elevation of AFP. Patients with an elevated AFP level are also classified as liver cirrhosis patients, in whom the lesion has remained nonmalignant for many years. At present, treatment options such as transcatheter chemoembolization, radiofrequency ablation, hepatectomy, and immunotherapy have not resulted in satisfactory clinical outcomes for HCC patients after intervention [81]. The high mortality rate of HCC is associated with the high rate of metastasis and recurrence [82]. At present, it is more common for HCC patients to be clinically diagnosed at an advanced stage [83]. Moreover, the effectiveness of the targeted therapy is only approximately 30% in patients with advanced HCC [84]. Therefore, exploring potential molecular changes in HCC cells to find effective therapeutic targets is necessary to address the current problem. Earlier studies have been found that LMNB1 is a detectable protein present in the plasma of HCC patients and is upregulated in HCC cells; thus, it could be measured as a potential diagnostic biomarker to identify patients who are in the initial stage of HCC [85]. In recent studies on HCC prognostic markers, Yang et al. [52] showed that LMNB1 overexpression in HCC cells was consistent with previous results. LMNB1 can promote HCC cell metastasis, increase HCC cell proliferation, and regulate the PI3K and MAPK signaling pathways [52]. Thus, high expression level of LMNB1 is also required by aggressive clinicopathological features of HCC and associated with low survival rate. Accurate prognostic assessment can earn critical time for appropriate treatment options; however, seldom studies have focused on the prognostic value of LMNB1 in HCC at present, and more studies are required on this topic. Compared with nonliver disease patients, Idriss et al. [86] conducted a research on 74 patients with liver disease and found that they had a higher expression level of LMNB1, as compared to patients with no liver disease; moreover, the LMNB1 level was significantly positively correlated with the AFP level. These results suggest that LMNB1 can be used as a potential marker for HCC. Abdelghany et al. [87] also reported similar results; the authors showed that the accumulated LMNB1 was released from HCC cells, thereby stimulating the p38 MAPK pathway and causing the cells to be in a state of oxidative stress. Additionally, the positive rate of LMNB1 mRNA was positively correlated with tumor stage [88]; this might be due to phospholipase C1-mediated LMNB1 phosphorylation; moreover, the intervention in the G2/M cell cycle process leads to cell proliferation, ultimately increasing the size and number of tumor tissues. The researchers suggest increasing the range of patients in whom LMNB1 levels should be measured to include patients with chronic liver disease with normal AFP levels, as these patients may experience rapid deterioration of their health condition.

### LMNB1 and renal carcinoma

Kidney cancer is one of the most common tumors of the urinary system, and it causes death of more than 170,000 people worldwide each year [55]. Clear-cell renal cell carcinoma (ccRCC) accounts for the majority of renal malignancies, and the incidence of ccRCC is increasing each year [58, 89]. Previous studies reported that gene inactivation (von Hippel-Lindau, VHL) was the primary factor for ccRCC tumorigenesis. The *VHL* gene shows somatic mutation in up to 80% of sporadic ccRCC cases and causes VHL disease, a familial cancer syndrome that predisposes affected patients to develop ccRCC [90]. In a recent study, Radspieler et al. [65] assessed tumor tissues of 763 ccRCC patients and found that LMNB1 expression was high in 80% of the analyzed ccRCC patients. During the aging process induced by etoposide, LMNB1 expression decreased, leading to a significant decrease in the proliferation rate of AKI-2, 786-O, and 769-P tumor cell lines. The authors suggested that LMNB1 could be used as a tissue-based biomarker to target new drugs for treating induced aging. However, few studies have investigated this aspect, and more clinical studies are required to further confirm the research results.

### LMNB1 and breast cancer

Breast cancer is one of the most common malignant tumors; it accounts for about 30 percent of new tumors in women and is the second leading cause of cancer-related death among women worldwide [58]. Recently, the rate of breast cancer patients with survival outcomes has improved significantly because of early screening, advances in molecular and pathological diagnosis, development of effective treatment modalities, and use of prognostic tumor markers such as CEA and CA125 [91]. However, further efforts are required to reduce morbidity and mortality associated with breast cancer. In particular, new drug targets and tumor markers may enable to predict the outcome of currently available therapies. Presently, the comparison of the expression level of LMNB1 in breast cancer tissues and normal breast epithelium has not received considerable attention. Earlier studies tended to correlate lower LMNB1 expression in breast cancer patients with poorer clinical outcomes, while higher LMNB1 expression levels are associated with better outcomes [46]. Setijono et al. [92] showed that miR-218 expression was downregulated in breast cancer cells, and the gene targeted by miR-218 was *LMNB1*. Saleh et al. [93] reported for the first time that the LMNB1 expression level was relatively high in both normal and malignant breast tissues, with an average positive expression rate of 93% and 88%, respectively; however, in malignant breast cancer cells exposed to neoadjuvant chemotherapy, LMNB1 expression was sharply reduced, with an average positive expression rate of 55%. In this study, however, the study population excluded stage IV breast cancer patients, and LMNB1 expression showed no prognostic value; therefore, the findings need to be verified by more representative studies. In recent years, membrane type 1-matrix metalloproteinase (MT1-MMP) has received increasing attention [94]. MT1-MMP is involved in extracellular matrix and basement membrane remodeling, thus enabling stability of LMNB1. MT1-MMP also promotes radiological and chemical resistance by destroying the extracellular matrix, which subsequently promotes restart of the replication fork [95]. Other studies have shown that chronic strain and stress can decrease LMNB1 expression levels and eventually led to DNA damage and nuclear membrane disruption; moreover, the release of cytoplasmic DNA activated the cGAS-STING signaling pathway that is dependent on cytoplasmic DNA response gene programs. This mechanically driven transcriptional rearrangement eventually changes the cell state, resulting in the malignant features of invasive breast cancer, including an epithelial-to-mesenchymal plasticity phenotype and chemotherapy resistance [96].

## Conclusion

As an important member of the lamin family, LMNB1 is involved in regulating various signaling pathways in cells, wherein it has key functions, including chromatin function regulation, cell cycle regulation, DNA repair, and response to oxidative stress. LMNB1 is also closely associated with the failure of normal cell cycle and the occurrence and development of various malignant tumors. Presently, LMNB1 is a hot research topic worldwide. Recent studies have shown that LMNB1 expression could serve as a potential marker of aging in melanoma [97] and could be utilized as a diagnostic reference for hematological malignancies [98]. However, the mechanism of action of LMNB1 remains unclear. LMNB1 expression and its prognostic value vary in different diseases; moreover, LMNB1 does not show the same expression pattern in the same disease [99]. Therefore, further investigations on the relationship between LMNB1 and cell senescence and malignant tumors are required to derive concrete results.

The present article systematically summarized and analyzed the recent progress in understanding the relationship between LMNB1 and cell senescence and malignant tumors. The review found that LMNB1 played a certain role in the regulation of cell senescence process as well as in the early screening, diagnosis, and treatment and prognostic assessment of malignant tumors. A limitation of this article is that few early LMNB1-related studies were reviewed. We believe that further in-depth research will enable to discover the mechanism of action of LMNB1, and related drugs targeting LMNB1 will gradually be used in clinical application, thus providing an important marker for disease evaluation, prevention, and treatment.
